# Integration of CO_2_ Capture and Mineral Carbonation by Using Recyclable Ammonium Salts

**DOI:** 10.1002/cssc.201000441

**Published:** 2011-07-15

**Authors:** Xiaolong Wang, M Mercedes Maroto-Valer

**Affiliations:** Centre for Innovation in Carbon Capture and Storage (CICCS), Energy and Sustainability Research Division, Faculty of Engineering, University of NottinghamUniversity Park, NG7 2RD (UK), Fax: (+44) 115-951-4115

**Keywords:** ammonium salts, carbon dioxide fixation, carbon storage, green chemistry, mineral carbonation

## Abstract

A new approach to capture and store CO_2_ by mineral carbonation using recyclable ammonium salts was studied. This process integrates CO_2_ capture with mineral carbonation by employing NH_3_, NH_4_HSO_4_, and NH_4_HCO_3_ in the capture, mineral dissolution, and carbonation steps, respectively. NH_4_HSO_4_ and NH_3_ can then be regenerated by thermal decomposition of (NH_4_)_2_SO_4_. The use of NH_4_HCO_3_ as the source of CO_2_ can avoid desorption and compression of CO_2_. The mass ratio of Mg/NH_4_HCO_3_/NH_3_ is the key factor controlling carbonation and the optimum ratio of 1:4:2 gives a conversion of Mg ions to hydromagnesite of 95.5 %. Thermogravimetric analysis studies indicated that the regeneration efficiency of NH_4_HSO_4_ and NH_3_ in this process is 95 %. The mass balance of the process shows that about 2.63 tonnes of serpentine, 0.12 tonnes of NH_4_HSO_4_, 7.48 tonnes of NH_4_HCO_3_, and 0.04 tonnes of NH_3_ are required to sequester 1 tonne of CO_2_ as hydromagnesite.

## Introduction

Carbon dioxide capture and storage (CCS) is considered to be one of the main solutions for reducing anthropogenic CO_2_. CCS includes CO_2_ capture from a point source, such as power plants, and transportation to a suitable site, where it is stored permanently and safely. Many projects for CO_2_ storage are based on direct injection of CO_2_ into underground formations (geologic sequestration) where it is stored by hydrodynamic, solubility, or mineral trapping.[1] However, the development of CO_2_ geological storage has been slow with respect to potential environmental impact and regulation for CO_2_ injection and monitoring.[2] Moreover, some countries, such as Finland and India, do not have sufficient storage capacity or lack suitable storage formations.[3] Therefore, there has been increasing interest in mineral carbonation.

The concept of CO_2_ sequestration by mineral carbonation is based on accelerating the weathering of rocks. CO_2_ reacts with alkaline earth oxide containing minerals to form insoluble carbonates. Magnesium and calcium silicate deposits, such as serpentine and olivine, can be used for this process. Due to the availability and abundance of these minerals, the capacity for mineral carbonation to store CO_2_ is estimated to be quite large.[4] Serpentine is an important source for this process due to its worldwide availability. For instance, a deposit of 30 000 km^3^ of magnesium silicates found in Oman would be able to store all of the anthropogenic CO_2_ generated from combustion of the world’s coal reserves.[5] Clearly, one of the main advantages of this process is the permanent safe storage of CO_2_ due to the thermodynamically stable nature of the solid carbonates formed.[4] Moreover, carbonation is an exothermic process, which may reduce the overall energy consumption and costs.[5]

However, the slow reaction rate of mineral dissolution is the main barrier to this process.[5] Many researchers have focused on promoting the dissolution rate by using different solvents, such as H_2_SO_4_, HCl, HNO_3_, organic acids, and inorganic salts.[3, 6–8] For example, Maroto-Valer et al. have reported 70 % dissolution of serpentine by using 2 m H_2_SO_4_ in 2 h.[6] A multistep aqueous carbonation process developed by Teir et al. used 4 m HCl or HNO_3_ to dissolve Mg ions from serpentine, then NaOH was used to control the pH of the solution to precipitate high purity hydromagnesite.[9] This process achieved 79 % carbonation efficiency at 80 °C and ambient pressure. They also reported that electrolysis of the NaCl solution was used to regenerate HCl and NaOH.[9] However, this process suffered from a high energy penalty in the regeneration process, in which the energy consumption for electrolysis of NaCl is 3277 and 4361 kWh t^−1^_CO_2_sequestered_ using HCl and HNO_3_, respectively.[9]

Therefore, there is a need to find low-cost, recyclable solvents that can provide high efficiency of mineral dissolution and carbonation. Recently, Krevor and Lackner tested NH_4_Cl, NaCl, sodium citrate, sodium EDTA, sodium oxalate, and sodium acetate to dissolve serpentine.[10] All experiments were carried out at 120 °C and 20 bar (1 bar=10^5^ Pa) CO_2_ in a batch autoclave. On using 0.1 m citrate, EDTA, and oxalate solutions, 60 % dissolution efficiency of magnesium from serpentine was achieved within 2 h, going up to 80 % after 7 h and reaching nearly 100 % between 10 and 20 h. Therefore, mineral dissolution with organic solvents is promising in terms of dissolution efficiency, but the dissolution rate is relatively slow. Pundsack reported the use of NH_4_HSO_4_ to dissolve serpentine and bubbled CO_2_ directly into the high-concentration magnesium solution obtained with aqueous ammonia to precipitate magnesium carbonates.[11] The dissolution efficiency of magnesium for this process was 92.8 %, but the carbonation efficiency was only 35 %. Fagerlund et al. proposed a process for the production of Mg(OH)_2_ from serpentine by using (NH_4_)_2_SO_4_.[12] A solid–solid reaction of serpentine with (NH_4_)_2_SO_4_ was carried out at >440 °C to generate MgSO_4_, which was added to aqueous ammonia to precipitate Mg(OH)_2_ and regenerate (NH_4_)_2_SO_4_. Mg(OH)_2_ was then carbonated with CO_2_ directly in a pressurised fluidised bed (PFB) reactor at 470–550 °C and 20 bar. However, only 20–60 % extraction efficiency of magnesium from serpentine was reported,[13] and the carbonation efficiency of Mg(OH)_2_ only achieved a maximum value of 50 %. This was due to the conversion of Mg(OH)_2_ into MgO at the temperature range used, at which the produced MgO cannot react with CO_2_ to produce carbonates.[14] Therefore, work is needed to improve both dissolution and carbonation efficiencies.

**Figure 1:**
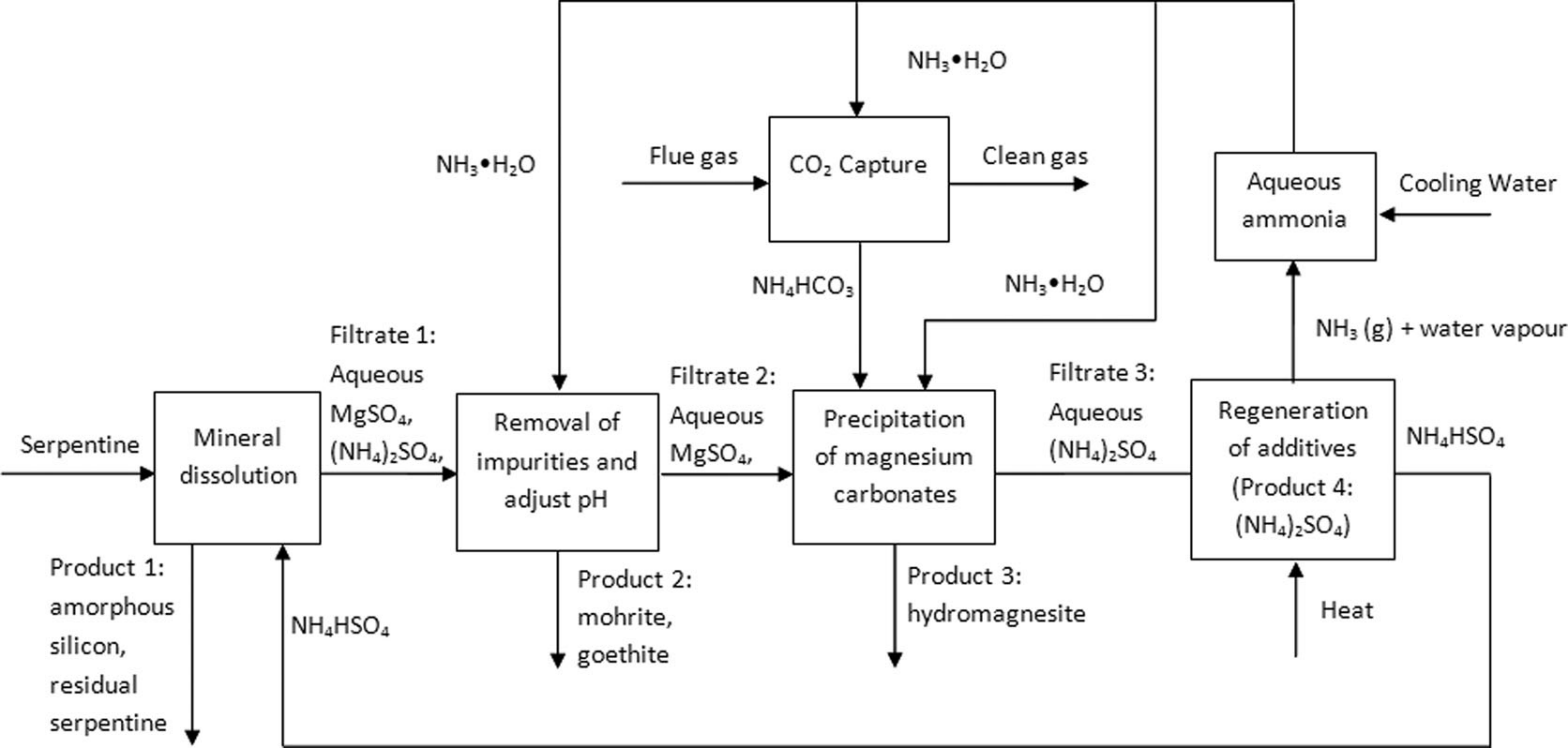
Modified process route of pH-swing CO_2_ mineral sequestration with recyclable ammonium salts.

We have developed a new pH-swing mineral carbonation process by using recyclable ammonium salts and the process route was presented in a previous paper.[15] The modified process diagram can be seen in Figure 1. In this process, aqueous NH_4_HSO_4_ was used to extract Mg from serpentine. The pH of the solution was then changed by adding aqueous ammonia, resulting in iron and silicon precipitating from solution. NH_4_HCO_3_ and NH_3_ were then added to the solution to react with Mg and produce carbonates and (NH_4_)_2_SO_4_, which was recycled from the solution by evaporation and then decomposed back into NH_3_ and NH_4_HSO_4_. Dissolution experiments of serpentine with NH_4_HSO_4_ have been previously reported.[15] It was found that 1.4 m NH_4_HSO_4_ could extract 100 % Mg from serpentine, as well as 98 % Fe and 17.6 % Si in 3 h at 100 °C. In addition, the dissolution kinetics of the reaction were found to follow the model of constant size particles with a rate-limiting control step of the chemical reaction by using product layer diffusion control.[15]

It must be pointed out that this process is unique in using NH_4_HCO_3_ instead of direct CO_2_ gas for mineral carbonation. The advantages of using NH_4_HCO_3_ include avoiding CO_2_ desorption in the capture step and subsequent CO_2_ compression for transportation, which are energy-intensive steps in the conventional CCS process.[15]

We have previously reported the dissolution of serpentine with NH_4_HSO_4_.[15] Herein, we investigated carbonation with NH_4_HCO_3_ and NH_4_HSO_4_ and NH_3_ regeneration. The carbonation experiments were conducted at different molar ratios of Mg/NH_4_HCO_3_/NH_3_. Finally, the mass balance of all streams in this process is presented.

## Experimental Section

### Preparation of magnesium salt solutions from serpentine using NH_4_HSO_4_

Previous dissolution experiments conducted by us have shown that NH_4_HSO_4_ is suitable for extracting magnesium from serpentine.[15] The chemical equation for dissolution of magnesium from serpentine using NH_4_HSO_4_ is presented in Equation 1:


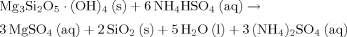
(1)

For the dissolution experiments, the same procedure and serpentine sample was used as in our previous paper.[15] Different temperatures (80, 90, and 100 °C) and reaction times (1, 2, and 3 h) were used for the preparation of solutions of MgSO_4_. After dissolution, the solution was cooled to room temperature and filtered by using 0.45 μm Pall syringe filters. The filtrate is referred to as filtrate 1 (Figure 1) and was used for the pH regulation studies described in the section below on pH regulation and removal of impurities. The solid residue was dried at 105 °C overnight and is referred to as product 1 (Figure 1). Filtrate 1 was analysed by inductively coupled plasma atomic emission spectroscopy (ICP-AES) to measure the concentration of dissolved Mg, Fe, and Si. For the purpose of this analysis, filtrate 1 was acidified with 70 wt % HNO_3_ to prevent precipitation of Mg and Fe. Product 1 was sampled and sent for X-ray fluorescence (XRF) analysis to determine the Mg, Fe, and Si content. The details about the instruments used for ICP, XRD and XRF, as well as the errors of these analyses, can be found elsewhere.[15]

### pH regulation and removal of impurities

About 40 % excess NH_4_HSO_4_ was used for the dissolution of serpentine to maximise magnesium extraction. After dissolution, the pH values of the solution were about 0.9–1.2. Because the carbonation reaction was favourable at high pH values, it was necessary to increase the pH of the solution to alkaline values. The chemical reaction of the pH regulation is presented in Equation (2):


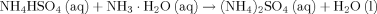
(2)

The reason for using aqueous ammonia is because the above reaction produces ammonium sulfate, which can be converted back into NH_3_ and NH_4_HSO_4_ in the regeneration step to recycle the additives (Figure 1).

If a high-value product (pure magnesium carbonate) is desired, some impurities, such as Fe, Al, Cr, Zn, Cu, and Mn, need to be precipitated from the system first by increasing the pH. To optimise the removal of impurities, extra aqueous ammonia was added to filtrate 1 after pH regulation, and the reactions for the removal of impurities are presented in Equations 3 and 4:


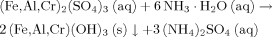
(3)


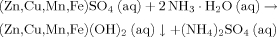
(4)

During pH regulation and removal of impurities, aqueous ammonia (35 wt %) was added to filtrate 1 until the pH value was neutral. During this process, the solution was stirred and an in situ pH probe was used to measure the pH value. The solution was filtered with 0.7 μm Pall syringe filters. The filtrate is referred to as filtrate 2 (Figure 1) and was used for the carbonation experiments described in the section below on precipitation of hydromagnesite using NH_4_HCO_3_. The solid residue was dried at 105 °C overnight and is referred to as product 2 (Figure 1). Filtrate 2 was analyzed by using ICP-AES to quantify the concentration of different elements, including Mg, Si, Fe, Mn, Zn, Cu, Al, and Cr. Product 2 was analysed by using XRF and XRD to quantify its composition and identify the mineral phases present.

### Precipitation of hydromagnesite using NH_4_HCO_3_

The reaction of precipitation of hydromagnesite by treating MgSO_4_ with NH_4_HCO_3_ and NH_3_ is presented in Equation 5:


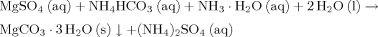
(5a)


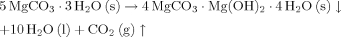
(5b)

The formation of magnesium carbonate species depends on temperature and pressure.[16] Nesquehonite (MgCO_3_**⋅**3 H_2_O) can precipitate from aqueous solutions at ambient temperatures as described in Equation 5 a, whereas at higher temperatures (50 and 100 °C), nesquehonite is transformed into hydromagnesite (4 mgCO_3_**⋅**Mg(OH)_2_**⋅**5 H_2_O), as presented in Equation 5 b. For temperatures above 100 °C, hydromagnesite is transformed into magnesite (MgCO_3_). In this study, hydromagnesite was produced because the experiments were conducted at 85 °C.

For the carbonation experiments, filtrate 2 was added to a 500 mL three-necked glass vessel and heated to 60 °C by using a silicon oil bath. The experimental setup was the same as that previously reported.[15] The time, temperature, and pH values were recorded every 5 min. Before heating, aqueous ammonia (35 wt %) was added to filtrate 2. When the temperature reached 60 °C, NH_4_HCO_3_ (as the CO_2_ source) was added and the solution was heated to 90 °C. After the solution was stabilised at 90 °C, the solution was kept at that temperature for 30 min. Aliquots (2 mL) were sampled by using a needle syringe at 5, 10, 15, 30, 45, and 60 min. The liquid samples were filtered by using a mini filter unit and acidified with HNO_3_ for subsequent ICP-AES analysis to measure the Mg concentration. At the end of the experiment, the solution was cooled and filtered by using 0.7 μm Pall syringe filters and the filtrate is referred to as filtrate 3 (Figure 1). The solid residue was dried at 105 °C overnight and is referred to as product 3 (Figure 1). The composition of product 3 was analysed by using XRF, and the mineral phases were identified by using XRD. Experiments were conducted at different mass ratios of Mg/NH_3_/NH_4_HCO_3_, in which Mg is the mass of Mg in filtrate 2, and NH_3_ and NH_4_HCO_3_ represent the mass of aqueous ammonia and the mass of NH_4_HCO_3_ added, respectively. In addition, a preliminary experiment was conducted in which no NH_3_ was added. The matrix of the experiments conducted at different mass ratios is listed in Table 1.

**Table 1:** Matrix of the molar ratios of Mg/NH_4_HCO_3_/NH_3_ and carbonation efficiency. For experiments 3 and 4, aqueous ammonia was added at ambient temperature, therefore, double ammonium magnesium was precipitated. For all the other experiments, aqueous ammonia was added as the temperature reached 60 °C

Experiment	Mg	Ratio NH_4_HCO_3_	NH_3_	Carbonation [%]
preliminary	1	3	0	25.5
1	1	3	1	71.6
2	1	3	0.5	53.0
3	1	3.5	1.5	46.5
4	1	4	1.5	53.4
5	1	2	1	41.5
6	1	4	1	77.9
7	1	5	1	89.9
8	1	4	2	95.9
9	1	4	1.5	91.5
10	1	4	3	91.3

The carbon content of product 3 was measured by using a thermogravimetric analysis (TGA) Q500 analyzer. The temperature programme was from 30 to 950 °C at 20 °C min^−1^ under a nitrogen atmosphere. The carbonation efficiency from soluble magnesium sulfate to hydromagnesite is defined by Equation 6:


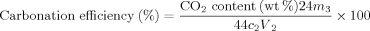
(6)

in which CO_2_ content (wt %) is the weight loss of product 3 during the temperature range from 300 to 500 °C, corresponding to the carbonate decomposition found in TGA studies;[17] *m*_3_ is the mass (g) of product 3 from carbonation experiment; *c*_2_ is the magnesium concentration in filtrate 2 from ICP-AES and *V*_2_ is the volume of filtrate 2, whereas 24 and 44 are the total molecular weights of Mg and CO_2_ in hydromagnesite, respectively.

### Thermal decomposition of (NH_4_)_2_SO_4_

Filtrate 3 was evaporated by using a rotary evaporator at 60 °C for 15 min. The solid collected from the rotary evaporator is referred to as product 4 (Figure 1). The regeneration of NH_4_HSO_4_ and NH_3_ was conducted by thermal decomposition of product 4 in an oven at 330 °C, and the reaction is presented in Equation 7:


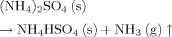
(7)

The thermal decomposition of product 4 was characterised by performing TGA studies by using a TGA Q500 instrument in the temperature range of 30–530 °C under nitrogen atmosphere. The temperature programme was as follows: from 30 to 230 °C at 10 °C min^−1^, hold for 10 min at 230 °C, up to 330 °C at 10 °C min^−1^, hold for 10 min at 330 °C, and finally up to 530 °C at 10 °C min^−1^. The choice of these three heating steps was to avoid decomposition of the mixture of products. To ascertain the decomposition from the TGA of product 4, pure (NH_4_)_2_SO_4_ and NH_4_HSO_4_ were also characterised by using TGA using the same heating procedure.

## Results and Discussion

### Preparation of magnesium salts solutions from serpentine using NH_4_HSO_4_

**Figure 2:**
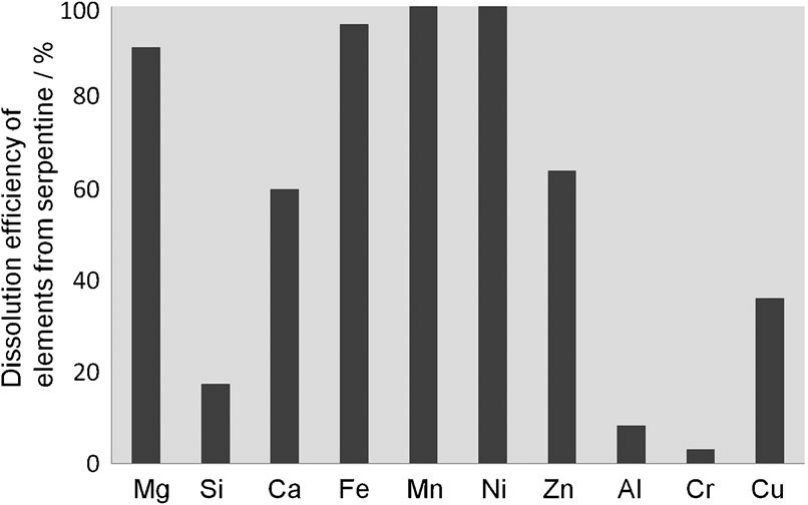
Dissolution efficiency of different elements after serpentine dissolution by using NH_4_HSO_4_ (experiment 3, 100 °C, 2 h).

The results from the ICP-AES analyses (Table 2) of the filtrate 1 solutions from all experiments show that high concentrations of Mg and Fe were dissolved, whereas most of the Si remained in the serpentine. The dissolution efficiency is calculated as the percentage of dissolved elements in filtrate 1 solution over elements in parent serpentine. The values for serpentine are also reported in Table 2. Taking experiment 3 as an example, using the data in Table 2, the dissolution efficiency of Mg from serpentine was 91 % for 1.4 m NH_4_HSO_4_ at 100 °C for 2 h. The dissolution efficiency of other elements for experiment 3 is presented in Figure 2. It was found that 96 % Fe, 17 % Si, 100 % Ni and Mn, and some Ca, Zn, Cu, and Al were also extracted from serpentine. This result is consistent with previous dissolution studies, in which the dissolution efficiencies of Mg, Fe, and Si from serpentine were 95, 83, and 17 %, respectively, under the same experimental conditions.[15] The dissolution efficiencies for Ni, Mn, Ca, Zn, Cu, and Al were very similar for all experiments conducted. Because high purity MgCO_3_ is desired, all other cations are considered to be impurities; Fe and Si are identified as the main impurities and are reported in Table 2. In conclusion, Mg was removed from serpentine, leaving behind amorphous silica. This could be explained by incongruent dissolution of Mg and Si, as previously discussed,[15] for which a chemical reaction with product-layer diffusion control was found to be the rate-limiting step of serpentine dissolution in NH_4_HSO_4_.

**Table 2:** Summary from ICP-AES analyses of parent serpentine and filtrate samples produced in the experiments

Sample	Filtrate	Mg [mg L^−1^]	Si [mg L^−1^]	Fe [mg L^−1^]	Dissolution [%]	Carbonation [%]
serpentine		11 970.0	3680.0	2050.0		
	1	8851.0	505.2	1179.0		
1	2	8252.0	74.1	600.0	73.8	71.56
	3	2347.0	11.2	1.0		
	1	9052.0	447.0	1284.0		
2	2	8290.0	110.0	244.0	75.4	53.0
	3	3219.0	20.2	0.5		
	1	10957	621.0	1964.0		
3	2	9629.0	98.0	337.0	91.3	46.5
	3	2983.0	5.2	0.3		
	1	8497.0	399.0	1131.0		
4	2	7840.0	105.0	187.0	70.8	53.4
	3	2914.0	21.0	0.2		
	1	9550.0	411.0	1133.0		
5	2	8690.0	154.0	145.0	79.6	41.5
	3	4097.0	35.2	0.9		
	1	8261.0	478.0	1175.0		
6	2	7866.0	132.0	206.0	68.8	77.9
	3	1625.0	19.4	0.2		
	1	8960.0	463.0	1084.0		
7	2	8123.0	98.0	133.1	74.7	89.9
	3	865.0	15.2	0.4		
	1	8487.0	422.0	1064.0		
8	2	7679.0	68.5	97.0	70.7	95.9
	3	889.2	6.9	0.6		
	1	6311.0	289.5	738.3		
9	2	5784.0	58.5	80.0	52.6	91.5
	3	589.8	12.5	0.3		
	1	8264.0	311.0	1135.0		
10	2	7794.0	82.5	98.0	68.9	91.3
	3	780.6	8.5	0.7		

Finally, to compare our work with that of Pundsack,[11] carbonation experiments were carried out by following his procedure. CO_2_ was bubbled into the prepared high-magnesium concentration solution from serpentine and excess aqueous ammonia was added to adjust the pH to a value of 9. Only 35 % carbonation efficiency was obtained. In comparison, the carbonation efficiency from this work can achieve a maximum of 95.9 % (experiment 8) due to the faster reaction rate between NH_4_HCO_3_ and Mg.

### pH regulation and removal of impurities

**Figure 3:**
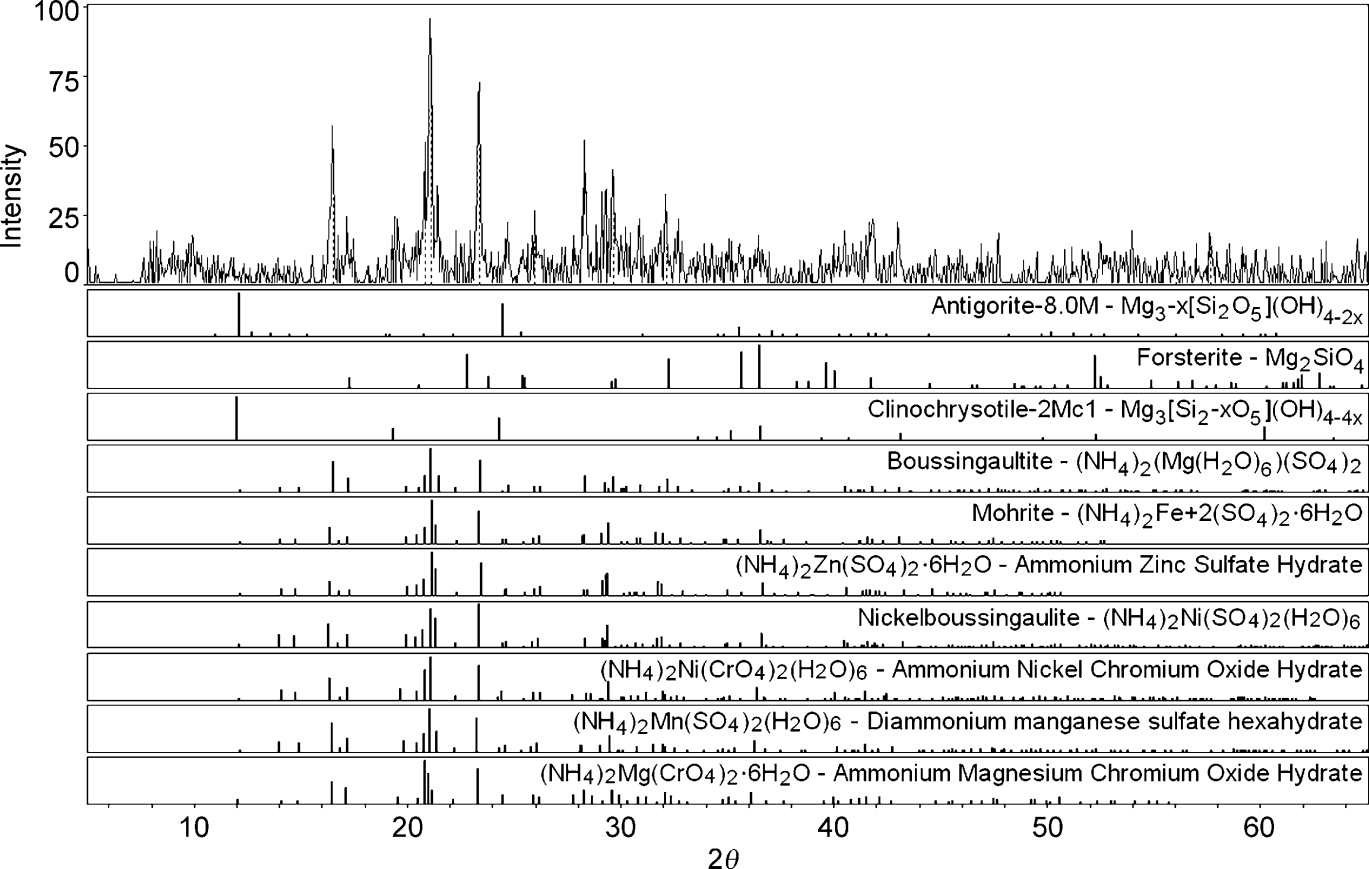
XRD pattern of product 2 from experiment 7.

**Figure 9:**
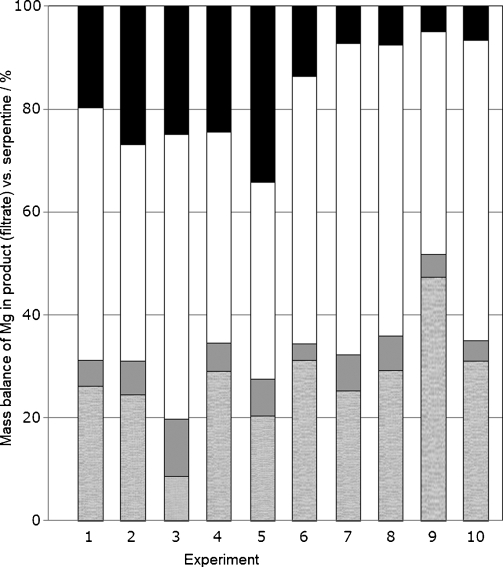
Mass balance of magnesium in products 1, 2, and 3 and filtrate 3 in relation to serpentine. From top to bottom: filtrate 3/serpentine (black), product 3/serpentine (white), product 2/serpentine (dark grey), and product 1/serpentine (light grey).

It was found that after adding aqueous ammonia (35 wt %) to filtrate 1 solution, some particles precipitated. After filtering and drying overnight at 105 °C, the resulting solid and filtrate were labelled product 2 and filtrate 2 (Figure 1), respectively. Aqueous ammonia (35 wt %) was then added to filtrate 2 until the pH value reached 8.5. Table 3 presents the XRF results of the products and the mass balance for Mg, Si, and Fe will be discussed in the section below on mass balance (Figure 9). Taking experiment 7 as an example, it can be seen that product 2 consists of 19.3 % Fe, 8.2% Si, and 2.8 % Mg. The XRD pattern of product 2 for experiment 7 (Figure 3) allowed the identification of double ammonium salts, (NH_4_)_2_Fe_2_(SO_4_)_2_**⋅**6 H_2_O, (NH_4_)_2_Mg_2_(SO_4_)_2_**⋅**6 H_2_O and (NH_4_)_2_Zn_2_(SO_4_)_2_**⋅**6 H_2_O, as the major phases. The presence of these double ammonium salts resulted from excess aqueous ammonia. Hot water flashing can decompose these double ammonium salts into ammonium sulfate and insoluble hydroxide salts.[18] Table 2 clearly shows that the concentration of Fe in filtrate 2 decreased significantly relative to filtrate 1. This decrease in Fe concentration indicates that Fe precipitates. The results of XRF, ICP-AES, and XRD analysis in Tables 1 and 2 and Figure 3 are consistent with this observation, indicating that a high iron-content precipitate was produced. Some magnesium also precipitated during this procedure, causing filtrate 2 to contain 5 % less dissolved magnesium than filtrate 1. All experiments presented similar XRF, ICP-AES, and XRD results for product 2 and filtrate 2. Moreover, the iron content of product 2 was measured by using XRF to be between 16.3 and 27.5 wt % (Table 3).

**3:** XRF analyses of solids produced in the experiments, and the CO_2_ content from TGA. The mass balance for Mg, Si and Fe for the three products in relation to parent serpentine is also presented as the mass ratio

Sample	Product	Mg [wt %]	Mass ratio [%]	Si [wt %]	Mass ratio [%]	Fe [wt %]	Mass ratio [%]	CO_2_[a] [wt %]
serpentine		23.9	100	22.2	100	5.7	100	N/A
	1	10.5	26.2	42.3	86.3	5.6	42.5	N/A
1	2	2.7	5.0	6.3	11.7	20.4	28.2	N/A
	3	21.5	49.2	0.2	1.7	2.2	29.2	38.8
	1	9.8	24.6	43.6	87.9	5.3	37.4	N/A
2	2	2.0	6.6	6.4	9.2	21.2	50.7	N/A
	3	16.5	42.3	0.3	2.4	0.8	11.9	30.1
	1	3.5	8.7	45.7	83.1	3.0	4.2	N/A
3	2	5.4	11.1	11.0	14.2	27.5	79.2	N/A
	3	21.9	55.4	0.2	2.5	1.1	16.4	39.5
	1	11.8	29.2	42.4	89.2	5.8	44.8	N/A
4	2	2.5	5.5	8.8	8.0	18.5	46.0	N/A
	3	20.1	41.1	0.3	2.3	0.8	9.1	37.5
	1	8.2	20.4	42.8	88.8	5.8	44.7	N/A
5	2	3.4	7.2	5.8	7.0	20.1	48.2	N/A
	3	23.8	38.3	0.4	3.2	0.8	7.0	40.9
	1	12.5	31.2	42.2	87.0	5.6	42.7	N/A
6	2	3.9	3.3	6.4	9.4	21.3	47.3	N/A
	3	19.8	52.0	0.4	3.1	0.7	1.0	35.5
	1	10.1	25.3	40.7	87.4	5.9	47.1	N/A
7	2	2.8	7.0	8.2	9.9	19.3	46.4	N/A
	3	20.5	60.5	0.3	2.3	0.4	6.2	36.7
	1	11.7	29.3	42.0	88.5	6.0	48.1	N/A
8	2	1.6	6.7	9.6	9.6	19.8	47.2	N/A
	3	17.7	56.6	0.2	1.7	0.3	4.7	26.51
	1	18.7	47.4	35.4	92.1	7.1	64.0	N/A
9	2	4.8	4.4	13.9	6.3	20.2	32.1	N/A
	3	18.2	43.3	0.1	1.3	0.3	3.9	27.0
	1	12.5	31.1	43.4	91.5	5.8	44.6	N/A
10	2	4.6	3.9	4.7	6.2	16.3	50.6	N/A
	3	21.3	58.5	0.1	2.0	0.3	4.7	38.5

[a] N/A=not applicable.

### Precipitation studies

**Figure 4:**
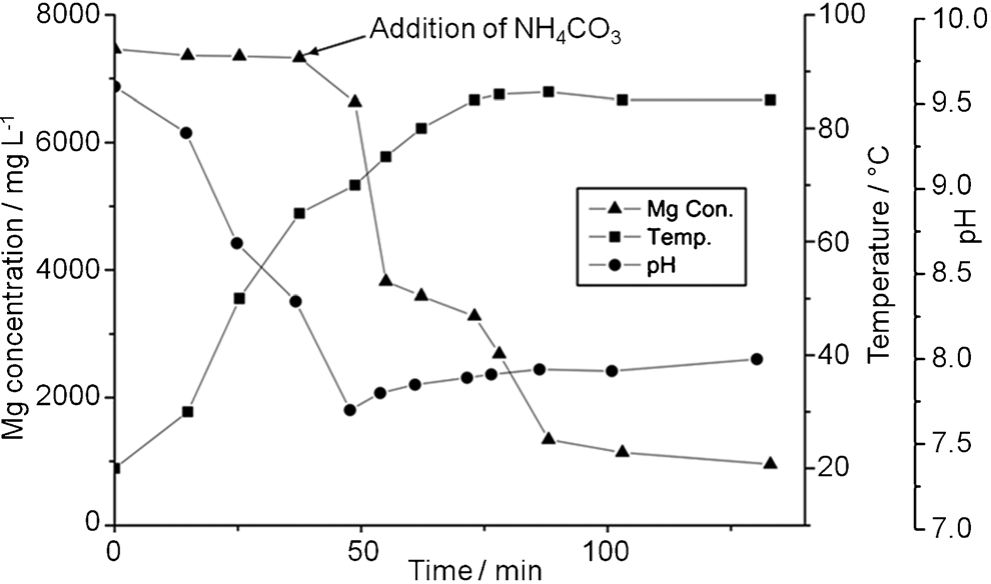
Temperature, time, pH, and concentration of magnesium in solution during the course of a typical carbonation experiment (experiment 7).

Ten precipitation experiments were carried out at different mass ratios of Mg/NH_3_/NH_4_HCO_3_, as shown in Table 1. The observations and findings from these ten experiments were similar in terms of carbonation and morphology of the products. Taking product 3 of experiment 7 as an example, Figure 4 shows the presence of magnesium carbonate. This corresponds to the decrease in magnesium concentration in solution for ICP-AES results presented in Table 2.

Figure 4 shows the magnesium concentration variation with time and temperature for experiment 7. The starting time was recorded as heating started. It can be seen that the pH of filtrate 2 decreased from 8.5 to 7.3 as the temperature increased during the first 20 min. As the temperature reached 60 °C, NH_4_HCO_3_ was added, and the pH increased slightly to 7.6. No precipitate was formed before adding NH_4_HCO_3_. The concentration of magnesium started to drop as the temperature went up to 70 °C at 25 min. In the following 5 min, half of the Mg ions precipitated at a very high rate of 33.3 mmol min^−1^. As the temperature stabilised at 85 °C after 40 min, the pH became stable, and Mg precipitated at a constant rate of 7.9 mmol min^−1^. 25 min after the addition of NH_4_HCO_3_, the concentration of Mg in solution became constant and finally fell below 1000 mg L^−1^.

**Figure 5:**
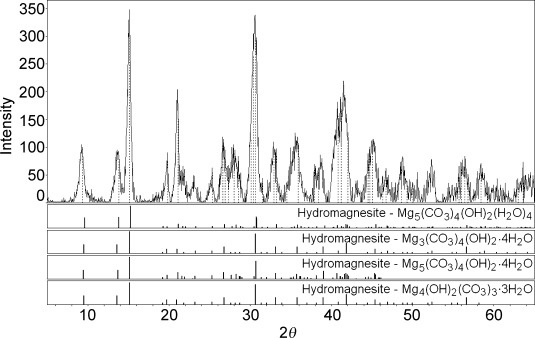
XRD pattern of product 3 of experiment 7.

For product 3 of experiment 7, the XRD pattern of product 3 (Figure 5) showed that the Mg precipitated as hydromagnesite, Mg_5_(CO_3_)_4_(OH)_2_**⋅**4 H_2_O. Combining the results from XRF of product 3 (Table 3) and the ICP-AES results from filtrate 3 (Table 2), it can be concluded that product 3 is 80 % pure hydromagnesite with only 0.79 wt % of Fe and 0.29 wt % Si.

**Figure 6:**
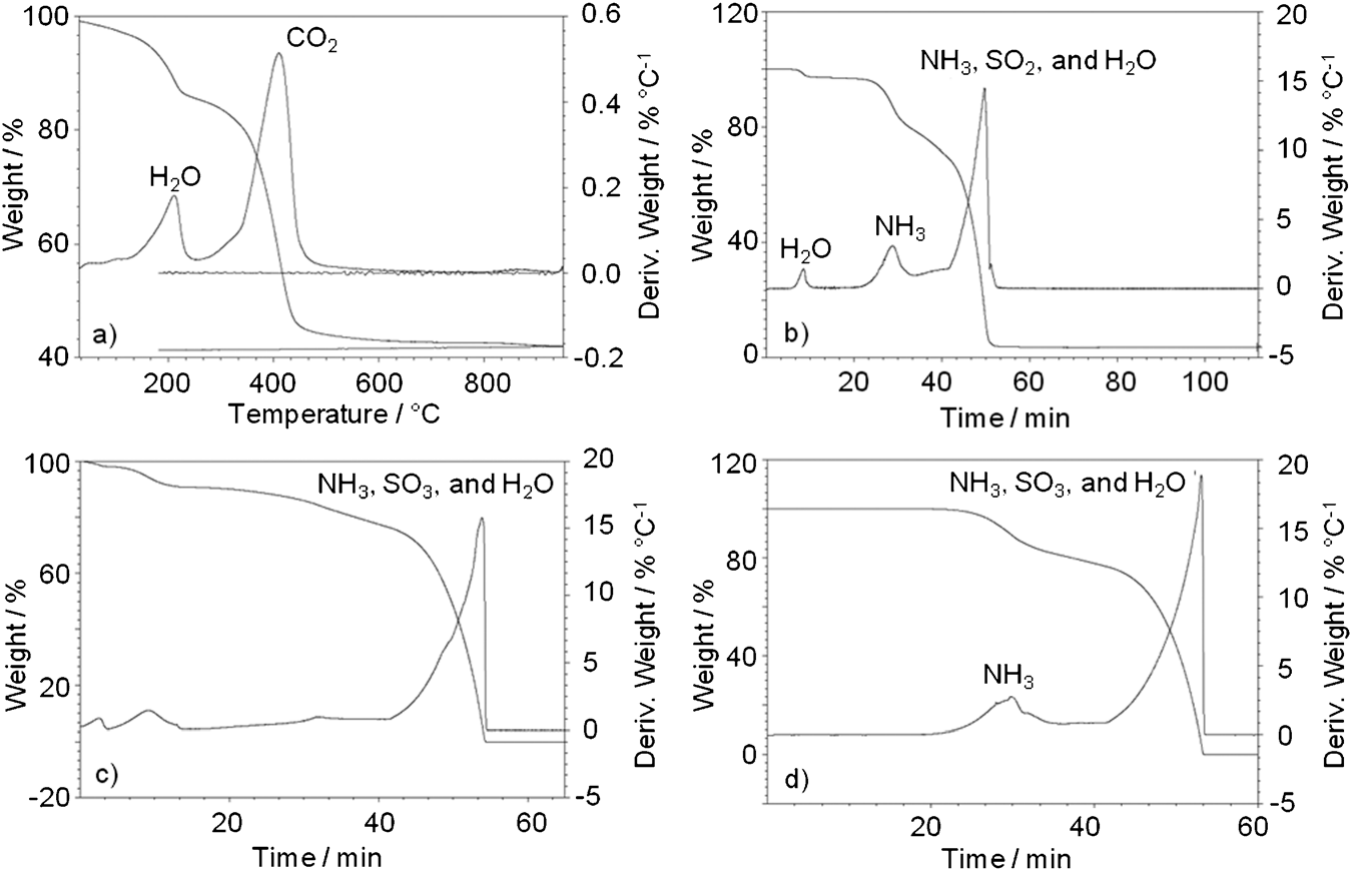
TGA profiles of a) product 3, b) product 4, c) NH_4_HSO_4_, and d) (NH_4_)_2_SO_4_ (products 3 and 4 were obtained in experiment 7).

The carbon content of product 3 can be calculated from the TGA profiles (Figure 6 a), as described in the Experimental Section. All samples presented one carbonate phase, according to the XRD studies. Therefore, the mass of the identified carbonate phase was estimated based on the corresponding weight loss from the TGA studies. As an example, Figure 6a shows two peaks: the first peak below 250 °C is about 12 wt % and corresponds to the release of crystal water,[17] the second peak, located between 250 and 500 °C, accounts for 37 wt % and is due to the decomposition of hydromagnesite.[16] Finally, based on the CO_2_ content (Table 3) and the Mg concentration in filtrate 2 (Table 2), it can be calculated that the carbonation efficiency of experiment 7 is 90 %.

### Improving carbonation by adding NH_3_

During the carbonation step, the Mg ions first react with HCO_3_^−^ to form Mg(HCO_3_)_2_ [Eq. 8 a], which then thermally decomposes into insoluble nesquehonite at above 70 °C, and then molecular MgCO_3_ is hydrated to form nesquehonite, followed by the transformation of nesquehonite into hydromagnesite [Eq. 5 b]. It must be pointed out that in the thermal decomposition reaction of Mg(HCO_3_)_2_ into MgCO_3_**⋅**3 H_2_O [Eq. 8 b], 1 mol of Mg(HCO_3_)_2_ is converted into 1 mol of insoluble MgCO_3_**⋅**3 H_2_O and 1 mol of CO_2_. This means that the maximum stoichiometric carbonation efficiency from soluble Mg(HCO_3_)_2_ into precipitated MgCO_3_**⋅**3 H_2_O is only 50 %. As an example, in the preliminary experiment in which no NH_3_ was used (Table 1), the carbonation efficiency was only 25.5 %. However, the joint use of aqueous ammonia and NH_4_HCO_3_ can improve the carbonation, as described by the reactions presented in Equations 9–14:


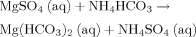
(8a)


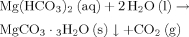
(8b)


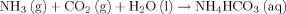
(9)


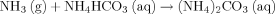
(10)


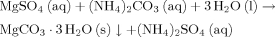
(11)


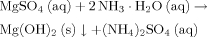
(12)


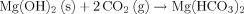
(13)


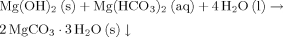
(14)

Ammonia captures CO_2_ to regenerate NH_4_HCO_3_ [Eq. 9]; this reaction is already used in CO_2_ capture technology.[19, 20] Ammonia can convert NH_4_HCO_3_ into (NH_4_)_2_CO_3_ [Eq. 10], which can directly produce MgCO_3_ [Eq. 11]. Ammonia can also react with MgSO_4_ to form insoluble Mg(OH)_2_ if the pH value is above 10, as shown in Equation 12. [21] Once the CO_2_ is released from the decomposition of Mg(HCO_3_)_2_ [Eq. 8 b], Mg(OH)_2_ can react with CO_2_ to form Mg(HCO_3_)_2_ [Eq. 13]. Moreover, Mg(OH)_2_ can also react with Mg(HCO_3_)_2_ directly to precipitate MgCO_3_ [Eq. 14]. Therefore, the carbonation efficiency was improved by the addition of aqueous ammonia to the high-magnesium concentration solution. In experiments 1–10, in which aqueous ammonia was added, the carbonation efficiency could reach up to 95.9 % (Table 1, experiment 8).

### Prevention of precipitation of magnesium ammonium carbonate

**Figure 7:**
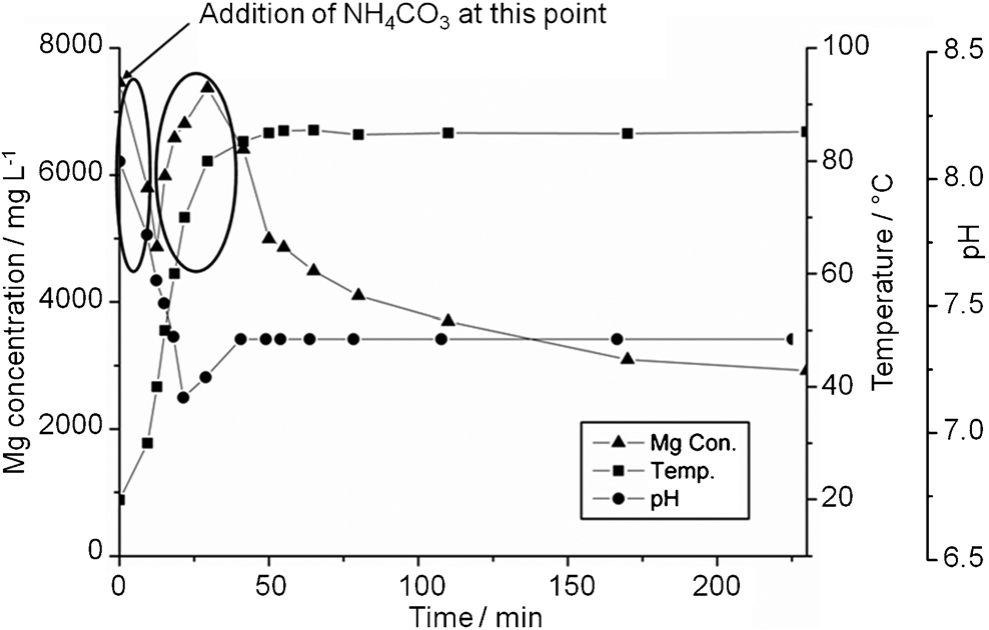
Temperature, time, pH, and concentration of magnesium in solution during the course of a carbonation experiment as double ammonium carbonate precipitates (experiment 4).

The precipitation of magnesium ammonium carbonate (MgCO_3_**⋅**(NH_4_)_2_CO_3_**⋅**4 H_2_O) can reduce carbonation efficiency because MgCO_3_**⋅**(NH_4_)_2_CO_3_**⋅**4 H_2_O is generated from the reaction of NH_3_ and NH_4_HCO_3_ with Mg ions at temperatures below 60 °C [Eq. 15].[22] It can be seen from Figure 7 that the magnesium concentration decreased until the temperature reached 60 °C during the first 15 min. However, MgCO_3_**⋅**(NH_4_)_2_CO_3_**⋅**4 H_2_O decomposes quickly to produce Mg(HCO_3_)_2_ and NH_3_ gas if the temperature goes above 60 °C [Eq. 16].


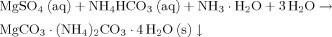
(15)


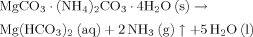
(16)

According to Equation 16, NH_3_ is produced, which would decrease the carbonation efficiency due to a shortage of NH_3_. Therefore, the precipitation of MgCO_3_**⋅**(NH_4_)_2_CO_3_**⋅**4H_2_O should be prevented to maintain high carbonation efficiency. Taking experiment 4 as an example, the precipitation of MgCO_3_**⋅**(NH_4_)_2_CO_3_**⋅**4 H_2_O is indicated on the top left corner of Figure 7. If the temperature increased above 60 °C, the Mg concentration increased, indicating the decomposition of MgCO_3_**⋅**(NH_4_)_2_CO_3_**⋅**4 H_2_O. The subsequent decrease of magnesium ions after 30 min indicates the precipitation of hydromagnesite. The carbonation efficiency of experiment 4 is as low as 53.4 % due to the shortage of NH_3_ gas, which escaped from the reaction system during the thermal decomposition of MgCO_3_**⋅**(NH_4_)_2_CO_3_**⋅**4 H_2_O. Comparing experiments 4 and 9 by using the same mass ratio of Mg/NH_4_HCO_3_/NH_3_ and same experimental conditions, the carbonation efficiency decreased from 91.5 to 53.4 % if there was precipitation of MgCO_3_**⋅**(NH_4_)_2_CO_3_**⋅**4 H_2_O (Table 1). Therefore, to prevent low carbonation efficiency caused by precipitation of magnesium ammonium carbonate, NH_4_HCO_3_ should preferably be added into the solution above 60 °C.

### Thermal decomposition of (NH_4_)_2_SO_4_

Product 4 is obtained from the carbonation step by evaporating filtrate 3 (Figure 1). Product 4 was used to generate NH_3_ and NH_4_HSO_4_ by thermal decomposition in an oven at 330 °C for 20 min. The released gas (NH_3_) was collected by using water to produce aqueous ammonia. The solid residue obtained after heating was NH_4_HSO_4_. These results were verified by conducting TGA studies, as described herein. Studies of the thermal conversion of ammonium sulfate into ammonium bisulfate can be found in several patents.[23–25] As an example in this work, the thermal decomposition of product 4 from experiment 7, as studied by TGA, is shown in Figure 6 b. The TGA profile shows two peaks, where the first weight loss below 330 °C is about 21.7 wt %, corresponding to the release of NH_3_ and the formation of NH_4_HSO_4_.[23–25] The second weight loss between 350 and 500 °C is 75.8 wt % and is due to further decomposition of NH_4_HSO_4_.[23–25] In total, the weight loss of product 4 is 97.5 wt % and the residual 2.5 wt % is due to the presence of MgSO_4_ that did not react during carbonation. The TGA profile of pure (NH_4_)_2_SO_4_ (purchased from Fisher Scientific) is presented in Figure 6 c, where two peaks appear at the same temperature range as those for the TGA profile of product 4 (Figure 6 b). The TGA curve of NH_4_HSO_4_ is presented in Figure 6 d and shows only one peak between 330 °C and 500 °C due to decomposition into NH_3_, H_2_O and SO_3_. The NH_4_HSO_4_ and NH_3_ regeneration efficiency from (NH_4_)_2_SO_4_ has been reported to be nearly 97 %.[23–25] Herein, the regeneration efficiency of NH_4_HSO_4_ and NH_3_ from product 4 is 95 %. These TGA results indicate that the reaction of thermal decomposition of (NH_4_)_2_SO_4_ should not be conducted above 330 °C to avoid further decomposition, because NH_4_HSO_4_ can decompose into NH_3_, SO_3_, and H_2_O above 330 °C.

### The effect of the mass ratio of Mg/NH_4_HCO_3_/NH_3_ on carbonation

The mass ratio of Mg/NH_4_HCO_3_/NH_3_ is the key factor controlling carbonation efficiency, as discussed herein. The stoichiometric mass ratio of Mg/NH_4_HCO_3_ is 1:2, but experiment 5 shows that if a stoichiometric ratio of 1:2 is used, the carbonation efficiency is only 41.5 % (Table 1). Increasing the Mg/NH_4_HCO_3_ ratio can improve the carbonation efficiency, as presented in Table 1, whereby the carbonation efficiency increases to 71.6, 77.9, and 89.9 %, if the ratio of Mg/NH_4_HCO_3_ is 1:3, 1:4, and 1:5, respectively. This can be explained by the thermal decomposition of NH_4_HCO_3_ [Eq. 17], and reported by Zhang et al.[19] NH_4_HCO_3_ can regenerate NH_3_ and release CO_2_ if the temperature is above 70 °C. The two reactions [Eq. 5 and Eq. 17] compete for NH_4_HCO_3_, and this may cause low carbonation efficiency due to the shortage of NH_4_HCO_3_.


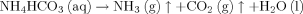
(17)

Moreover, adding aqueous ammonia can increase the carbonation efficiency, as discussed in the section above on precipitation studies. In comparison to the preliminary experiment, experiments 1 and 2 show that carbonation efficiencies increase from 25.5 % to 53 (experiment 2) and then 71.6 % (experiment 1) if the mass ratio of Mg/NH_4_HCO_3_/NH_3_ increases from 1:3:0 to 1:3:0.5 and then 1:3:1, respectively. This trend was also found in experiments 6, 8, and 9; however, if the ratio increases to 1:4:3, the carbonation efficiency does not increase any further.

**Figure 8:**
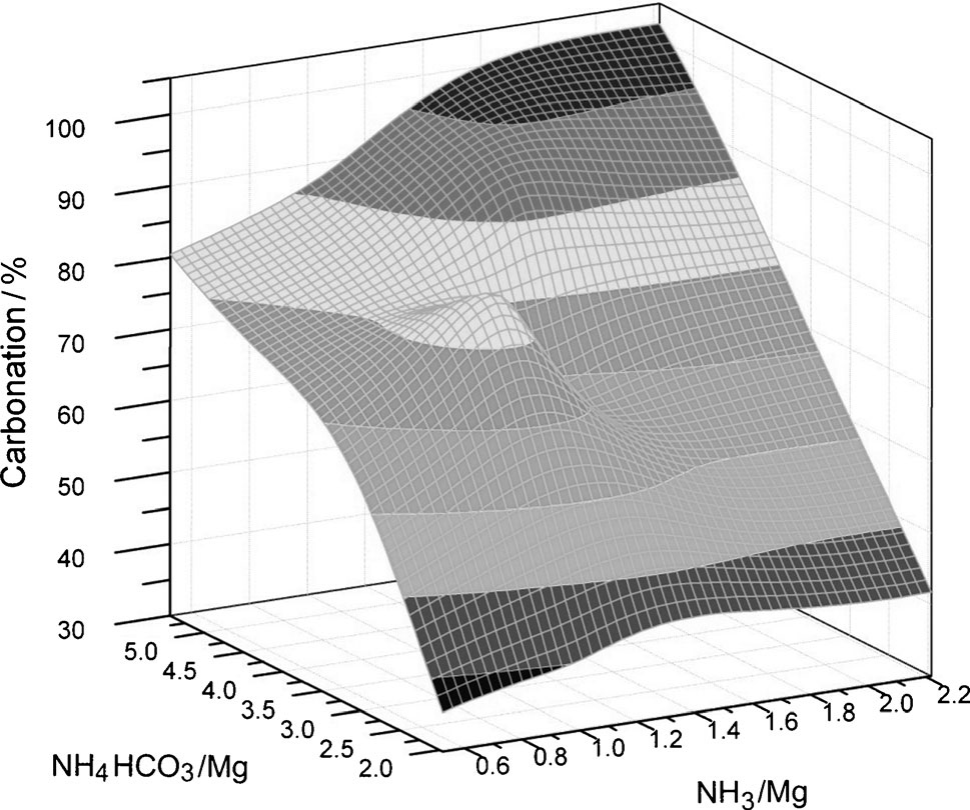
Carbonation efficiency versus NH_4_HCO_3_/Mg and NH_3_/Mg molar ratio.

Herein, the optimum mass ratio of Mg/NH_4_HCO_3_/NH_3_ was determined. A 3D graph (Figure 8) is used to show the relationship of the four variables, including mass of Mg, mass of NH_4_HCO_3_, mass of NH_3_, and carbonation efficiency. Figure 8 clearly shows that a low summit of 71.6 % carbonation efficiency appears if the mass ratio of Mg/NH_4_HCO_3_/NH_3_ is 1:3:1 and a high summit of 95.9 % carbonation efficiency appears if the mass ratio of Mg/NH_4_HCO_3_/NH_3_ is 1:4:2. Continuously increasing both NH_4_HCO_3_ and NH_3_ does not result in a further significant rise of the carbonation efficiency. However, an optimum amount of NH_4_HCO_3_ and NH_3_ are needed to achieve the highest carbonation efficiency due to the loss of CO_2_ and NH_3_ in an open system.

The process studied herein presents higher carbonation efficiency than that reported previously.[8, 21] For example, in a work by Gerdemann et al.,[8] 64 % carbonation efficiency was achieved in direct carbonation of heat-treated serpentine at 155 °C and 115 bar CO_2_ in 0.64 m NaHCO_3_ and 1 m NaCl solution. In a work by Teir et al.,[21] the conversion of Mg ions into hydromagnesite was 94 % using HNO_3_ and 79 % using HCl at pH 9 with the addition of NaOH (1.1 g NaOH/g precipitate). Herein, the highest carbonation efficiency is 95.9 % at 85 °C and ambient pressure within 30 min by using NH_4_HCO_3_ and NH_3_.

### Mass balance

To examine the distribution of magnesium released from serpentine in the solids formed in the process (products 1, 2, and 3) and filtrate 3, a mass balance was constructed based on the XRF and ICP-AES and the results are presented in Figure 9. It can be seen that most of the magnesium from the parent serpentine ends up in the precipitated hydromagnesite (product 3). The use of additives at the optimised ratio to improve carbonation conversion results in less magnesium remaining in the final solution after carbonation (filtrate 3, experiments 6–10). Longer dissolution times may leach more magnesium from the serpentine and therefore reduce the amount present in product 1.[7] In addition, the presence of magnesium in product 2 can be minimised by hot-water washing.[18] The mass balance for Si and Fe is presented in Table 3, as the concentration of these two elements in filtrate 3 is very small. It can be seen that most of the Si remains in the residue after dissolution (product 1). In contrast, most of the Fe ends up in both the residue after dissolution (product 1) and the precipitate after pH swing (product 2), depending on the dissolution efficiency. The concentration of Si and Fe in filtrate 3 is negligible (Table 2). The mass balance for the three elements studied (Mg, Si, and Fe) is very good and between 99–100 % of the mass of the three elements is accounted for.

Considering that the dissolution efficiency can reach 90 % at 100 °C after 2 h and that the carbonation efficiency is 95.9 % if the molar ratio of Mg/NH_4_HCO_3_/NH_3_ is 1:4:2, the net conversion of serpentine to hydromagnesite is calculated to be 86.3 %. Taking this into account, about 2.63 tonnes of serpentine, 8.48 tonnes of NH_4_HSO_4_, 7.48 tonnes of NH_4_HCO_3_, and 0.8 tonnes of NH_3_ are required to sequester 1 tonne CO_2_, and 2.95 tonnes of hydromagnesite is produced. If 95 % regeneration efficiency of NH_4_HSO_4_ and NH_3_ is considered, 0.12 tonnes of NH_4_HSO_4_ and 0.04 tonnes of NH_3_ are consumed to sequester 1 tonne CO_2_. All of the chemicals used in this process can be obtained from (NH_4_)_2_SO_4_. Considering that the current price for (NH_4_)_2_SO_4_ is 90 US$ tonne^−1^,[26] the cost of chemicals in this process is estimated to be 18 US$ tonne^−1^_CO_2__. However, in a work by Teir et al., the cost of chemicals is 1300 US$ tonne^−1^_CO_2__ using HCl and 1600 US$ tonne^−1^_CO_2__ using HNO_3_.[21] Moreover, the cost could be brought down further by using high solid/liquid ratios and this will be the focus of future work.

## Conclusions

We have studied the precipitation of hydromagnesite from prepared high-magnesium concentration solutions by using NH_3_ and NH_4_HCO_3_. The regeneration of NH_3_ and NH_4_HSO_4_ was also investigated. Pure hydromagnesite can be produced from serpentine by using regenerated ammonium salts with a net conversion of 86.3 %. Amorphous silica can be obtained from the dissolution step. Byproducts with a maximum 27.5 wt % iron content were obtained from the pH regulation and removal of impurities step. The additives used, NH_4_HSO_4_ and NH_3_, can be regenerated by thermal decomposition of (NH_4_)_2_SO_4_ at 330 °C. The addition of aqueous ammonia before carbonation could significantly improve the carbonation efficiency. It must be pointed out that NH_4_HCO_3_ should be added to the solution after 60 °C to prevent the production of magnesium ammonium carbonate. The mass ratio of Mg/NH_4_HCO_3_/NH_3_ was the key factor to control the carbonation efficiency, and it was found that if the mass ratio of Mg/NH_4_HCO_3_/NH_3_ was 1:4:2, a carbonation efficiency of 95.9 % was achieved. From the TGA studies, the regeneration efficiency of NH_4_HSO_4_ in this process was found to be 95 %. According to mass balance, about 2.63 tonnes of serpentine, 0.12 tonnes of NH_4_HSO_4_, 7.48 tonnes of NH_4_HCO_3_, and 0.04 tonnes of NH_3_ are required to sequester 1 tonne_CO_2__, and 2.95 tonnes of hydromagnesite is produced.
